# Identification by mass spectrometry and automated susceptibility testing from positive bottles: a simple, rapid, and standardized approach to reduce the turnaround time in the management of blood cultures

**DOI:** 10.1186/s12879-017-2851-5

**Published:** 2017-12-06

**Authors:** Carola Mauri, Luigi Principe, Silvia Bracco, Elisa Meroni, Nicoletta Corbo, Beatrice Pini, Francesco Luzzaro

**Affiliations:** 1 Microbiology and Virology Unit, A. Manzoni Hospital, Lecco, Italy; 2 Present address: Clinical Pathology Unit, Vimercate Hospital, Vimercate, Italy

**Keywords:** Bloodstream infection, Time to positivity, Processing time, Rapid identification, MALDI-TOF, VITEK 2

## Abstract

**Background:**

Speeding up identification and antimicrobial susceptibility testing (AST) is of foremost importance in the management of blood cultures. Here, we describe a simple, rapid, and standardized approach based on a very short-term incubation on solid medium from positive blood cultures followed by MALDI-TOF mass spectrometry identification and automated AST. The aim of the study was to evaluate the impact in the laboratory practice of this new procedure with respect to that previously used (standard method) by comparing TAT and cumulative percentage of final reports to clinicians.

**Results:**

Compared with the standard method, the new procedure gave correct organism identification at genus or species level in 98.4% of monomicrobial samples. AST resulted in 97.7% essential agreement and 98.1% categorical agreement, with 0.9% minor errors, 1.0% major error, and 1.5% very major errors. The mean turnaround time to identification and AST was 61.4 h by using the new method compared to 83.1 h by using standard procedure. Concerning cumulative percentages of final reports, approximately a third of results were available at 48 h from the check-in of the sample when using the new procedure, whereas no final reports were ready at the same time with the standard method.

**Conclusions:**

The new procedure allows faster and reliable results using a simple and standardized approach. Thus, it represents an important tool for a more rapid management of blood cultures when molecular methods are not available in the laboratory.

## Background

Rapid identification and antimicrobial susceptibility results of organisms causing bloodstream infections (BSIs) are an absolute priority for the Microbiology Laboratory. Blood culture is the gold standard method for the diagnosis of BSIs, and it is included among the early activities in the Surviving Sepsis Campaign guidelines [1]. However, it is time-consuming and usually takes on average 24–48 h for microbial growth in blood culture bottles, and further 48 h for identification and antimicrobial susceptibility tests after growth in solid culture media [2]. Speeding up these procedures is of foremost importance in the management of bloodstream infections, leading to rapid administration of adequate antimicrobial therapy or adjusting ongoing treatment, and so improving outcome in patients with bacteremia [3]. Reporting Gram stain results of positive blood cultures is useful to guide empiric antimicrobial therapy, but does not provide information about microbial identification at species level [4]. Molecular methods such as real-time polymerase chain reaction (RT-PCR), fluorescent in-situ hybridization (FISH), DNA microarray and nested multiplex PCR have shown to be efficient for the identification of specific microorganisms [5, 6]. However, molecular methods and related target-based multiplex assays can identify a limited range of microorganisms and antimicrobial resistance genes. Furthermore, molecular methods require additional hands-on processing time and costs [7, 8].

Matrix-Assisted Laser Desorption Ionization Time-of-Flight Mass Spectrometry (MALDI-TOF MS) technology has been introduced as a way to quickly and accurately identify bacteria and yeast [9, 10]. Compared to standard phenotypic identification, this technology is rapid, inexpensive (after initial purchase of the instrument), and can identify bacteria grown on solid media to the species level [11–13]. Subsequent to the introduction of MALDI-TOF MS for routine diagnostic microbiology, several applications have been developed to further improve and accelerate pathogen identification, especially for bloodstream infections [14, 15]. Particularly, several authors investigated the possibility of direct identification of bacteria from positive blood cultures with MALDI-TOF MS [7, 8, 16–20] while fewer investigated the combined use of MALDI-TOF MS and automated antimicrobial susceptibility testing (AST) instruments [3, 21, 22]. Also, direct identification and AST performed on positive blood culture using mass-spectrometry and disk diffusion methods, respectively, can provide results about 24 h earlier than routine standard methods. However, disk diffusion for direct susceptibility testing has not been fully standardized, because it is not related to a specific quantification of the bacterial concentration of the inoculum.

At our Institution, a number of specific approaches have been used to reduce the time of result and improve patient outcome: delivery of blood cultures to the laboratory within one hour, immediate incubation of bottles in the automated instrument and continuous management of blood cultures (365 days/year), communication of preliminary results based on Gram stain (performed at the time of positivity), identification from overnight cultures by MALDI-TOF MS, and direct AST by agar diffusion (using selected antimicrobials as indicators of resistance).

This paper describes a standardized, easy-to-perform and practical approach in order to obtain rapid identification with MALDI-TOF MS and to decrease the time needed to obtain final reports using a standardized bacterial concentration and an automated instrument for definitive susceptibility results. The turnaround time (TAT) was also compared to the standard method to better quantify the impact in the laboratory practice. The aim of the study was to compare these data with those obtained with the previously used workflow of blood cultures.

Part of this work has been presented at the 25th ECCMID meeting, Copenhagen (Denmark), 2015 (ePoster EP-054).

## Methods

### Study design

The study was organized in two subsequent steps. In the first period, conducted from January to October 2014, positive bottles from blood cultures obtained from different patients were investigated using the current standard routine method in parallel with the new procedure.

In the second period, conducted from November 2014 to September 2015, the new procedure was introduced as the routine method, except for polymicrobial blood cultures. TAT resulting in the second period (i.e., using the new procedure as routine method) was then evaluated in comparison with that obtained in the first period (i.e., using the previous standard method).

Concerning internal workflow, our laboratory runs 24/7 but routine microbiological procedures are performed from 8 am to 5 pm. Blood cultures, however, are delivered from wards to the laboratory within one hour from collection and are immediately incubated in the automated instrument at any time (i.e. 24/7). Positive bottles are routinely processed every day within the opening time of the laboratory. Of note, the new procedure was applied 5 days/week (Monday-Friday), from 8 am to 2 pm and only one positive blood culture for each patient was processed. Blood cultures that were found positive from 2 pm to 5 pm were only processed by the standard routine method. Because of limited laboratory staff resources during evening and night hours (from 5 pm to 8 am), samples detected positive during this time were processed the following morning with the new procedure.

### Standard identification and antimicrobial susceptibility testing

Blood samples were inoculated in aerobic and anaerobic non-charcoal based bottles (BacT/ALERT® FA Plus, and FN Plus, respectively), containing adsorbent polymeric beads to neutralize antimicrobials, and incubated in the BacT/ALERT 3D system (bioMérieux, Marcy l’Etoile, France). The standard method for identification and susceptibility testing was performed as described: a small volume of positive blood culture fluid was inoculated on different plates, including Columbia agar with 5% sheep blood, Chocolate agar with polyvitex, Columbia ANC agar with 5% sheep blood, Mac Conkey agar, Schaedler agar with Vitamin K1 and 5% sheep blood, and Sabouraud agar with gentamicin and chloramphenicol. The inoculated plates were incubated at 36 °C in O2, 5% CO2 or anaerobic atmosphere to enable bacterial growth. Gram staining was performed at the time of blood culture positivity detection and immediately communicated by phone to the treating physicians.

Identification was done by MALDI-TOF MS (VITEK® MS, bioMérieux). Isolated colonies from agar plates were directly applied to VITEK MS target slide in duplicate (two spots for each isolate) and were covered with one microliter of CHCA (α-Cyano-4-hydroxycinnamic acid) matrix. The identification was considered valid if at least one spot on the target slide gave a confidence level of ≥75% without conflicting identifications from replicate spots of the same sample. A standardized inoculum (McFarland standard of 0.5) was then prepared from single colonies grown on the agar medium, and the appropriate VITEK AST cards (AST-632 for staphylococci, AST-586 for enterococci, AST-01 for streptococcal species other than *Streptococcus pneumoniae*, AST-576 for *S. pneumoniae*, AST-202 for Gram-negative bacteria), chosen according to the identification results given by the VITEK MS, were inoculated following the manufacturer’s recommendations. Antimicrobial susceptibility results were obtained after a 18-24 h incubation using the VITEK® 2 (bioMérieux) instrument and interpreted according to current EUCAST criteria [23, 24].

### Rapid identification and antimicrobial susceptibility testing subsequent to very short-term incubation on solid medium

When a blood culture was flagged positive by the BacT/ALERT, an aliquot (2.5 ml) of each sample was transferred in a tube with gel separator (BD Vacutainer® Blood Collection Tubes) and centrifuged at 3500 rpm for 10 min. The supernatant was discarded and the pellet was inoculated on two blood agar plates and incubated at 36 °C in O2 and 5% CO2, respectively. After 3 h incubation, microbial identification was obtained by VITEK MS directly from bacterial growth on agar plates, as above described. Susceptibility tests were performed even when the rapid identification was available at genus level only. Concurrent with rapid identification, a suspension, adjusted to a McFarland standard of 0.5, was created from bacterial growth and used for AST on the VITEK 2 instrument as above described.

### Data analysis

Identification and susceptibility results obtained by the new procedure were compared with those obtained with routine standard method. Identification results were classified as correct identification at species or genus level, no identification, and incorrect identification. To compare susceptibility testing results, MICs obtained by both methods were translated in clinical categories as susceptible (S), intermediate (I), or resistant (R) according to the current EUCAST criteria and evaluated in terms of Categorical Agreement (CA), Essential Agreement (EA), Very Major Error (VME, false susceptibility), Major Error (ME, false resistance), and minor Error (mE, susceptible/resistant versus intermediate susceptibility) (International Standard ISO 20776–2) [25]. The Etest method (bioMérieux) was used to resolve interpretation discrepancies.

### Application of the new method and evaluation of the turnaround time

Following the application of the new method, the TAT, calculated as the time from the check-in of the sample to the final report comprehensive of identification and antimicrobial susceptibility test results, was evaluated for the two study periods. It consisted of two components: i) time to positivity, corresponding to the time necessary for the microorganism growth by BacT/ALERT incubation; and ii) processing time, corresponding to the time necessary for generating the final report (including bacterial identification, AST, validation of results, and reporting to clinicians). Cumulative percentages of final reports were also evaluated at different times starting from the check-in of the sample.

Times were calculated using the Microbiology software package (Copernico, bioMérieux) interfaced with the Laboratory Information System (Magellano, Software Team), and evaluated separately for Gram-positives and Gram-negatives.

## Results

### First study period

Two hundred and ten blood cultures obtained from different patients were examined during the first period. Compared with standard method, the new procedure allowed to obtain a correct identification at the species level in 187 (89.0%) cases (96 Gram-positives and 91 Gram-negatives), whereas 3 (1.4%) isolates were identified at the genus level. Identification at the genus level was obtained for *Streptococcus agalactiae* (*n* = 1), *Salmonella* group B (*n* = 1), and *Salmonella typhi* (*n* = 1). The isolate identified as *Salmonella* group B was not further investigated at the species level. Overall data are summarized in Table 1. No incorrect identifications were obtained.

**Table 1:** Distribution of blood isolates identified in the two study periods

Microorganism	First study period	Second study period
Gram-positive		
* S. aureus*	30	29
* S. epidermidis*	25	31
CoNS	16	13
* Enterococcus spp.*	16	12
* Streptococcus spp.*	10	12
Total Gram-positive isolates	97	97
Gram-negative		
* E. coli*	63	68
* K. pneumoniae*	10	8
* P. aeruginosa*	7	3
* A. baumannii*	–	2
Other Enterobacteriaceae	13	12
Total Gram-negative isolates	93	93
Total of isolates	190	190

Among the 97 Gram-positives, 71 belonged to the genus *Staphylococcus*, 16 were identified as *Enterococcus* spp., and 10 were identified as *Streptococcus* spp.. Of the 93 Gram-negatives, 86 belonged to the family Enterobacteriaceae and 7 were identified as *Pseudomonas aeruginosa*.

Of the remaining 20 blood cultures, 13 (6.2%) showed polymicrobial growth, and 3 (1.4%) were regarded as false positives (no growth). VITEK MS was unable to generate an acceptable identification (insufficient peaks) using the new procedure in 4 (1.9%) cases. Isolates not identified were: *Staphylococcus hominis* (*n* = 2), *S. agalactiae* (*n* = 1), and *Staphylococcus capitis* (*n* = 1). Overall, the new procedure gave correct organism identification at genus or species level in 98.4% of monomicrobial samples.

Only microorganisms identified at species or genus level were studied for AST. A total of 2462 microorganism-antimicrobial combinations were analyzed. Overall, results were as follows: EA, 97.7% (2405/2462); CA, 98.1% (2414/2462); mE, 0.9% (21/2462); ME, 1.0% (20/1940); and VME, 1.5% (7/478).

Results for Gram-positive bacteria were as follows: EA, 96.9%; CA, 97.4%; mE 0.8%; ME 1.7%; and VME, 2.5% (Table 2). Notably, VME were detected only among coagulase-negative staphylococci (CoNS). Results for Gram-negative isolates were: EA, 98.4%; CA, 98.7%; mE, 0.9%; and ME, 0.5% (Table 3). No VME were detected.

**Table 2:** Comparison of AST results for Gram-positives: agreement of the new rapid procedure with standard method

Antimicrobial agent	No. of test	Susceptibilities			EA		CA		mE		ME		VME	
		S	I	R	No.	%	No.	%	No.	%	No.	%	No.	%
Ampicillin	20	18	0	2	20	100	20	100	0	0.0	0	0.0	0	0.0
Ampicillin-sulbactam	16	14	0	2	16	100	16	100	0	0.0	0	0.0	0	0.0
Cefotaxime	9	9	0	0	9	100	9	100	0	0.0	0	0.0	0	0.0
Ceftriaxone	9	9	0	0	8	89	9	100	0	0.0	0	0.0	0	0.0
Clindamycin	91	52	0	39	80	88	82	90	2	2.2	6	11.5	1	2.6
Daptomycin	71	70	0	1	69	97	70	99	0	0.0	1	1.4	0	0.0
Erythromycin	95	48	1	46	93	98	89	94	5	5.3	1	1.1	0	0.0
Gentamicin	71	46	0	25	70	99	70	99	0	0.0	0	0.0	1	4.0
Imipenem	21	20	0	1	21	100	21	100	0	0.0	0	0.0	0	0.0
Levofloxacin	95	54	1	40	95	100	94	99	1	1.1	0	0.0	0	0.0
Linezolid	92	92	0	0	91	99	92	100	0	0.0	0	0.0	0	0.0
Nitrofurantoin	15	15	0	0	15	100	15	100	0	0.0	0	0.0	0	0.0
Oxacillin	70	36	0	34	70	100	70	100	0	0.0	0	0.0	0	0.0
Penicillin	81	23	0	58	78	96	80	99	0	0.0	0	0.0	1	1.7
Rifampicin	76	69	0	7	74	97	75	99	0	0.0	0	0.0	1	14.3
Teicoplanin	87	71	0	16	80	92	82	94	0	0.0	2	2.3	3	18.7
Tetracycline	76	49	15	12	75	99	74	97	1	1.3	1	1.3	0	0.0
Tigecycline	87	87	0	0	87	100	87	100	0	0.0	0	0.0	0	0.0
Vancomycin	97	97	0	0	91	94	93	96	0	0.0	4	4.1	0	0.0
TOTAL	1179	879	17	283	1142	96.9	1148	97.4	9	0.8	15	1.7	7	2.5

**Table 3:** Comparison of AST results for Gram-negatives: agreement of the new rapid procedure with standard method

Antimicrobial agent	No. of test	Susceptibilities			EA		CA		mE		ME		VME	
		S	I	R	No.	%	No.	%	No.	%	No.	%	No.	%
Amikacin	93	81	7	5	93	100	92	99	1	1.1	0	0.0	0	0.0
Amoxicillin-clavulanate	92	61	0	31	91	99	89	97	0	0.0	3	4.9	0	0.0
Cefepime	93	72	7	14	89	96	90	97	3	3.3	0	0.0	0	0.0
Cefotaxime	93	64	0	29	90	97	93	100	0	0.0	0	0.0	0	0.0
Ceftazidime	93	68	6	19	90	97	91	98	2	2.2	0	0.0	0	0.0
Ciprofloxacin	93	68	0	25	93	100	93	100	0	0.0	0	0.0	0	0.0
Colistin	89	85	0	4	87	98	89	100	0	0.0	0	0.0	0	0.0
Gentamicin	93	84	0	9	93	100	93	100	0	0.0	0	0.0	0	0.0
Ertapenem	93	79	1	13	93	100	93	100	0	0.0	0	0.0	0	0.0
Imipenem	89	86	0	3	89	100	89	100	0	0.0	0	0.0	0	0.0
Meropenem	93	90	1	2	93	100	93	100	0	0.0	0	0.0	0	0.0
Piperacillin-tazobactam	92	79	3	10	88	96	88	96	3	3.3	1	1.3	0	0.0
Tigecycline	84	79	2	3	83	99	81	96	3	3.6	0	0.0	0	0.0
Co-trimoxazole	93	65	0	28	91	98	92	99	0	0.0	1	1.5	0	0.0
TOTAL	1283	1061	27	195	1263	98.4	1266	98.7	12	0.9	5	0.5	0	0

Microorganism-antimicrobial combinations that did not result in agreement with conventional methods are listed in Table 4. No discrepancies were observed as regards detection and/or suggestion of the following resistance mechanisms: ESBL production (18/63 *Escherichia coli* were found to be consistent with ESBL producers); carbapenemase production (2/10 *Klebsiella pneumoniae* produced KPC-type carbapenemases); methicillin resistance (8/30 *Staphylococcus aureus* and 25/41 CoNS isolates were methicillin-resistant). With regard to inducible clindamycin resistance (ICR) in staphylococci, 10/30 *S. aureus* and 7/41 CoNS (*S. hominis*, *n* = 5; *Staphylococcus epidermidis*, *n* = 2) were ICR positive with both methods. Three disagreements were observed in CoNS (*S. epidermidis*, *n* = 2, and *S. hominis*, *n* = 1). In two cases, ICR test was positive with the new procedure but negative with the standard method whereas in the remaining case ICR test was negative with the new procedure but positive with the standard method. The D test consistently confirmed results of the standard method.

**Table 4:** Microorganism-antimicrobial combinations that did not result in agreement with standard method^a^

Microorganism	mE	ME	VME
*E. cloacae*	Tigecycline (*n* = 1)	–	–
*E. coli*	Piperacillin-tazobactam (*n* = 3);	Amoxicillin-clavulanate (*n* = 3);	–
	Cefepime (*n* = 2);	Piperacillin-tazobactam (*n* = 1);	
	Ceftazidime (*n* = 2);	Co-trimoxazole (*n* = 1)	
	Amikacin (*n* = 1)		
*E. faecalis*	–	Vancomycin (*n* = 1)	–
*K. pneumoniae*	Tigecycline (*n* = 2)	–	–
*P. mirabilis*	Cefepime (*n* = 1)	–	–
*S. aureus*	Erythromycin (*n* = 1);	Clindamycin (*n* = 2);	–
	Tetracycline (*n* = 1)	Daptomycin (*n* = 1);	
		Vancomycin (*n* = 1)	
*S. capitis*	Erythromycin (*n* = 1)	Clindamycin (*n* = 1);	–
		Teicoplanin (*n* = 1);	
		Tetracycline (*n* = 1);	
		Vancomycin (*n* = 1)	
*S. epidermidis*	Clindamycin (*n* = 2);	Clindamycin (*n* = 2);	Teicoplanin (*n* = 2);
	Erythromycin (*n* = 1)	Teicoplanin (*n* = 1)	Penicillin (*n* = 1);
			Rifampicin (*n* = 1)
*S. hominis*	Levofloxacin (*n* = 1)	Clindamycin (*n* = 1)	Clindamycin (*n* = 1);
			Gentamicin (*n* = 1);
			Teicoplanin (*n* = 1)
*S. pneumoniae*	Erythromycin (*n* = 2)	Erythromycin (*n* = 1);	–
		Vancomycin (*n* = 1)	

^a^Some isolates have more than one error. Microorganisms not listed did not have any microorganism-antimicrobial combination errors

### Second study period

One hundred and ninety blood cultures were evaluated during the second period (i.e., after implementing the new method in the laboratory routine). All of them were identified at the species level.

Among the 97 Gram-positives, 73 (75.3%) belonged to the genus *Staphylococcus*, 12 (12.4%) were identified as *Enterococcus* spp., and 12 (12.4%) were identified as *Streptococcus* spp.. Of the 93 Gram-negatives, 88 (94.6%) belonged to the family Enterobacteriaceae and 5 (5.4%) were identified as non-fermentative Gram-negatives. Distribution of isolates in this group was similar to that of isolates examined during the first period (Table 1).

### Evaluation of the turnaround time

The mean TAT values were calculated for the two periods in order to compare results obtained using the new procedure with respect to those obtained with the standard method, and are listed in Table 5. The time to positivity was comparable in the two study periods (16.0 h and 16.1 h, using standard and new method, respectively). Using the new approach, the overall mean TAT substantially decreased (from 83.1 h to 61.4 h), especially in the case of Gram-negatives (from 80.4 h to 56.7 h).

**Table 5:** Mean value of times in the two study periods. First quartile, median and third quartile are reported in brackets

Times (in hours)	Standard method			New procedure		
	Total	GP^a^	GN^a^	Total	GP	GN
Time to positivity	16.0	17.9	14.1	16.1	18.9	13.3
	(10.1–14.0-19.1)	(12.1–17.0-22.1)	(10.0–12.0-15.0)	(11.0–13.0-18.1)	(12.1–16.1-22.0)	(10.0–11.1-14.0)
Processing time	67.1	67.7	66.3	45.3	47.0	43.4
	(52.8–61.2-78.5)	(52.0–59.2-76.2)	(54.6–62.8-83.4)	(31.7–37.7-51.8)	(30.9–37.5-51.9)	(31.9–37.8-51.3)
Intra-laboratory TAT	83.1	85.6	80.4	61.4	65.9	56.7
	(69.3–74.7-93.8)	(69.2–74.8-89.7)	(69.5–74.5-95.6)	(47.6–51.4-72.5)	(48.6–59.8-73.1)	(44.6–50.5-65.6)

^a^GP: Gram-positives; GN: Gram-negatives

Table 6 shows cumulative percentages of final reports available at 48 and 72 h using the two methods. Of note, no final reports were ready at 48 h from the check-in of the sample using the standard method, whereas 26.5% of results were available at the same time using the new procedure. At 72 h, 37.3% and 74.6% of results were available using the standard method and the new procedure, respectively. *E. coli* and *S. aureus*, i.e., the most frequently isolated organisms for the two groups of Gram-negative and Gram-positive bacteria, were separately analyzed. In both cases cumulative percentages of final reports available at 48 and 72 h using the new procedure were higher than 30% and 80%, respectively.

**Table 6:** Cumulative percentages of final reports available at 48 and 72 h from the check-in

Microorganism	48 h		72 h	
	SM^a^	NP^a^	SM	NP
Total	0	26.5	37.3	74.6
Gram-positive	0	19.8	36.8	71.9
Gram-negative	0	33.3	37.8	79.6
*S. aureus*	0	32.1	28.6	82.1
*E. coli*	0	38.2	37.3	83.8

^a^SM: standard method; NP: new procedure

## Discussion

The present study describes a procedure based on a very short-term incubation time on solid medium from positive blood cultures followed by MALDI-TOF MS identification and automated AST (starting from a standardized bacterial concentration) in order to obtain rapid final results.

An early basis for decision making is urgently required in the case of life-threatening conditions such as bloodstream infections [26]. Of note, the International Guidelines for Management of Sepsis and Septic Shock recommend that: i) antimicrobial therapy should be assessed daily for de-escalation; ii) the combination therapy, when used empirically in patients with sepsis, should not be routinely used for ongoing treatment.

Empiric antimicrobial therapy should be narrowed once pathogen identification and sensitivities are established and/or adequate clinical improvement is noted [1]. Molecular methods have shown to be efficient for the rapid identification of specific microorganisms but can identify a limited range of microorganisms and antimicrobial resistance genes, thus partially responding to the above recommendations. Furthermore, molecular methods are not available in many laboratories, require additional hands-on processing time and costs. Application of MALDI-TOF MS for identification of bacterial colonies from solid media has considerably improved and accelerated routine microbiological diagnostics [11–13]. Particularly, direct MALDI-TOF MS pathogen identification from positive blood culture has been demonstrated to have a positive impact on antimicrobial treatment in septic patients [27, 28]. Most of procedures with this direct approach, however, require additional hands-on processing time. For example, Romero-Gómez et al. used a series of centrifugation, washes and an extraction procedure before identification and AST [3]. Machen et al. used a combined lysis-filtration method [21] whereas Prod’hom et al. used a series of centrifugation, washes and lysis procedure [29]. One major advantage of our combined method is its simplified workflow. Our procedure can be easily integrated in the laboratory routine since it needs only a tube with gel separator (as additional consumable) and a centrifugation step. Identification by MALDI-TOF MS and suspension for AST are performed following a common standard procedure, only earlier than usually (i.e., 3 h versus overnight incubation).

With respect to rapid identification, our results demonstrate that the performance of the presented method is very high and satisfactory for both Gram-positive and Gram-negative isolates. These data confirm the capacity of the MALDI-TOF MS technique to identify both Gram-positive and Gram-negative bacteria from positive blood cultures after a very short-term incubation time, as previously reported [18].

AST also showed excellent results. Percentages of errors (minor, major and very major) were overall low enough to fulfill the performance criteria considered acceptable by the International Standard ISO 20776–2 (ME ≤3%; VME ≤ 3%). According to previous observations, CoNS exhibited the majority of errors among Gram-positives [3, 21]. These strains, however, are common contaminants of blood cultures that often do not require an antimicrobial treatment. Consequently, the clinical impact of these errors is very low. Among Gram-negative isolates, *E. coli* had the highest rate of errors but no very major errors occurred. The large number of isolates could have contributed to this result. Similarly, our results indicate a high concordance with regard to detection and/or suggestion of known resistance mechanisms (e.g., production of ESBLs or carbapenemases, methicillin-resistance and ICR). Taken together, these data demonstrate that the rapid method is reliable not only for wild-type isolates (as previously suggested) [3] but also for multidrug-resistant microorganisms.

Finally, in addition to high performance rates for identification and AST, the new procedure exhibited TATs significantly lower than those obtained using the standard method. It is worth noting that the time to positivity was comparable in the two study periods (thus eliminating a confounding variable between the two groups). The processing time was substantially reduced using the new procedure, leading to a final result approximately one day earlier than usual. In this regard, the reduction of TAT was mainly influenced by the automated AST, performed the same day of bottle positivity, even though the application of MALDI-TOF MS technique was crucial to render it possible.

## Conclusions

The new procedure, based on rapid identification by MALDI-TOF MS and automated susceptibility testing performed following short-term incubation cultures on solid agar plates inoculated from positive blood cultures, represents a simple, standardized, and workflow-friendly approach that allows faster and reliable results essentially without additional hands-on processing time and costs. The most relevant conclusion from our experience is that the new procedure permits at least a one day reduction in TAT in the management of bloodstream infections, especially when the most frequently isolated pathogens are involved. The introduction of this method in the microbiology laboratory might facilitate an appropriate species-specific therapy with potential downstream impact on the development of resistance and improved patient outcomes.

## Abbreviations

AST: Antimicrobial susceptibility testing
BSI: Bloodstream infections
CA: Categorical agreement
EA: Essential agreement
MALDI-TOF MS: Matrix-assisted laser desorption ionization time-of-flight mass spectrometry
ME: Major error
mE: minor error
TAT: Turnaround time
VME: Very major error
